# Effects of self-efficacy on frontal midline theta power and golf putting performance

**DOI:** 10.3389/fpsyg.2024.1349918

**Published:** 2024-04-08

**Authors:** Chien-Lin Yu, Cheng-Wei Kao, Jia-Hao Wu, Eric Hung, Wei-Chun Chang, Ren-Ting Yang, Kuo-Pin Wang, Tsung-Min Hung

**Affiliations:** ^1^Department of Physical Education and Sport Sciences, National Taiwan Normal University, Taipei, Taiwan; ^2^Center for Cognitive Interaction Technology (CITEC), Bielefeld University, Inspiration 1, Bielefeld, Germany; ^3^Neurocognition and Action, Biomechanics Research Group, Faculty of Psychology and Sports Science, Bielefeld University, Bielefeld, Germany; ^4^Institute for Research Excellence and Learning Science, National Taiwan Normal University, Taipei, Taiwan; ^5^Lifestyles of Health and Sustainability Executive Master of Business Administration, National Taiwan Normal University, Taipei, Taiwan

**Keywords:** self-efficacy, frontal midline theta, attention, sports performance, golf-putting

## Abstract

**Introduction:**

Self-efficacy (SE), defined as an individual's belief in their ability to complete a task, is linked to top-down attentional control, influencing motor performance in sports. Although the behavioral effects of SE are well-documented, there is a lack of research on the mechanisms through which SE affects sports performance. Our research aims to elucidate the neurophysiological mechanisms that underlie the impact of self-efficacy on sports performance. Specifically, we intend to explore the effects of low and high SE on frontal midline theta (Fmθ) activity, associated with sustained top-down attention, and on motor performance.

**Methods:**

We recruited thirty-four professional golfers to perform 60 putts, during which their electroencephalographic activity was monitored. SE levels were assessed using a visual analog scale from 0 to 10 before each putt, with scores categorized into higher or lower SE based on each golfer's individual average score.

**Results:**

Paired *t*-tests indicated that trials with higher SE scores had a higher putting success rate than those with lower SE scores (53.3% vs. 46.7%). Furthermore, trials associated with higher SE scores exhibited lower Fmθ activity compared to those with lower SE scores (4.49 vs. 5.18).

**Discussion:**

Our results suggest that higher SE is associated with reduced top-down attentional control, leading to improved putting performance. These findings support Bandura's theory of SE, which suggests that the effects of efficacy beliefs are mediated by cognitive, motivational, emotional, and decision-making processes. This study sheds light on the intermediate processes of SE by examining its impact on the anticipation of outcomes, sports performance, and attentional control prior to putting.

## Highlights

- Identifying the cognitive-motor processes of superior performance can provide crucial information not only for accelerating the motor learning process but also for enhancing motor performance.
- Putting with higher SE was followed by less top-down attentional control, a characteristic of automatic processing, leading to better putting performance.
- Our findings support Bandura's SE theory, which posits that processes by which efficacy beliefs produce their effects are mediated by cognitive, motivational, emotional, and selection processes.
- The findings indicate that attentional control is a potential mediator of the relationship between SE and sports performance.

## 1 Introduction

Self-efficacy (SE) refers to individuals' belief in their abilities to complete a task or master a situation successfully (Bandura, 1997). In addition, SE can be considered situationally specific self-confidence, especially in sports (Feltz, 1988, 2007). Previous studies have recognized SE as a main determinant of successful performance (Moritz et al., 2000). Theoretical studies and meta-analyses have determined a strong relationship between SE and sports performance. For example, a meta-analysis of 41 studies demonstrated a moderate correlation between SE and sports performance, with an average correlation coefficient of 0.25 (95% confidence interval [CI]: 0.19, 0.30); the studies analyzed had minimal evidence of publication bias (Lochbaum et al., 2022). In addition, empirical studies have indicated that SE resulting from the successful practice of a motor task predicts performance on subsequent motor learning tests (Stevens et al., 2012; Pascua et al., 2015). The increased expectations of learners regarding their future successful performance can lead to even greater success, improvement, and learning (Rosenqvist and Skans, 2015). Moreover, compared with other psychological factors, SE can more effectively predict performance in high-level competitions (Ercis, 2018).

SE theory assumes that SE exerts a positive and significant effect on the performance of athletes. According to this theory, the mechanisms underlying the effects of efficacy beliefs or perceptions regarding SE on the outcomes of interest may be mediated by cognitive, motivational, emotional, and selection processes (Bandura, 1997). Various studies have demonstrated that higher SE can affect attention priming and prioritization, leading to increased attention to task-relevant cues and decreased attention to less relevant clues (Themanson and Rosen, 2015). Studies have identified an association of higher SE with increased attention to task error cues (Themanson et al., 2008) as well as higher response accuracy and faster reaction time during more difficult or incongruent task conditions (Themanson and Rosen, 2015). When action is planned and executed, higher performance expectancy may act as a buffer or protect against responses that would hinder optimal performance, such as nonbeneficial alternate responses, including off-task activities (Jiao et al., 2015; Zahodne et al., 2015). However, limited empirical studies have genuinely tested the theory (Lippke, 2020), especially the intermediate processes, such as cognitive, motivational, emotional, and selection processes, through which SE affects performance. Interestingly, most of the aforementioned studies have focused only on performance in cognitive tasks. The findings from these studies may not be generalizable to sports performance, as the required task success rates and movement patterns may differ.

Preaction top–down attentional control may play a key role in the relationship between SE and sports performance. According to the OPTIMAL (Optimizing Performance through Intrinsic Motivation and Attention for Learning) theory (Wulf and Lewthwaite, 2016), it explains the mechanisms underlying the association of SE with sports performance. This theory proposes that enhanced performance expectancy and prevented or reduced self-focus (or other off-task activity) contribute to effective goal-action coupling by preparing the motor system for task execution (Wulf and Lewthwaite, 2016; Lewthwaite and Wulf, 2017). High performance expectations appear to prepare the performer for successful movements through their impacts on attention and cognition, thus ensuring that objectives are effectively aligned with desired actions. However, the neurophysiological mechanisms underlying the association between SE with sports performance remain unclear. Therefore, identifying attentional control during preaction can provide critical information for optimizing performance and the benefits of SE.

Research in the field of motor performance has indicated that skilled performance can be “defined by high levels of automaticity, minimum energy expenditure, and reduced movement times” (Schmidt and Lee, 2014; Vickers and Williams, 2017, p. 5; Filho et al., 2021). An number of neuroscience studies recently have used electroencephalography, which can provide a high temporal resolution of neural activity, to investigate and identify the neurophysiological processes underlying athletic performance. For example, recent research in self-paced sports observed a significant decrease in Fmθ activity in experts compared to novices (Filho et al., 2021). Although athletes need to engage and disengage different areas of their brains to perform at optimal levels (i.e., brain proficiency), their frontal lobe works at the lowest rate possible (i.e., transient hypofrontality), which may explain the reported feelings of automaticity, control, confidence, and relaxation experienced by skilled performers when performing at optimal levels (Williams and Krane, 2020). This preaction hypofrontality (lower Fmθ) indicates decreased attention and working memory, which easily facilitate automatic actions (Dietrich et al., 2003; Dietrich, 2006) and sport performance in experts (Chen et al., 2022). The hypofrontality phenomenon may illustrate the process through which SE improves sports performance and explain the relationship between SE and changes in attention. Specifically, higher self-efficacy (SE) may influence pre-action attentional regulation, as suggested by Themanson and Rosen (2015), with decreased Fmθ brain activity being linked to sport performance. Consequently, Fmθ brain activity could act as a precise indicator for clarifying the mechanisms through which SE impacts sports performance.

**Figure 1:**
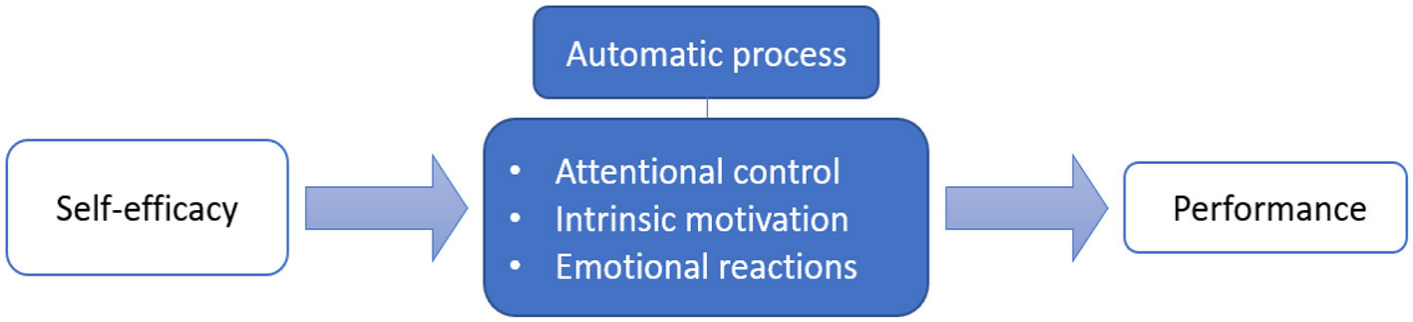
Conceptual framework.

Integrating the above theories, the OPTIMAL theory of sports performance can be supported by the principles of Self-Efficacy (SE) theory (Bandura, 1997; Themanson and Rosen, 2015). SE may enhance sports performance by enhancing expectations of outcomes, integrating goals and actions through prioritized attention, and inhibiting attention to less relevant task (as depicted in Figure 1). However, research on the neurophysiological mechanisms underlying the SE effects of sports performance is sparse. To provide new insights into the neurophysiological mechanisms underlying the effects of SE on sports performance, the present study utilized EEG and investigated the effect of low and high SE on Fmθ activity that involves with top–down sustained attention and on the motor performance in skilled golfers. To facilitate EEG recording, we utilized a golf putting task that necessitates maintaining motionlessness during data collection (Wang et al., 2019, 2020). Considering that individuals with high SE may possess automatic characteristics, such as reduced top–down attentional control and working memory (as depicted in Figure 1), leading to improved motor performance, we hypothesize that higher SE is associated with enhanced golf-putting performance and lower Fmθ power.

## 2 Methods

### 2.1 Participants

The study follows a Cross-sectional design, 34 right-handed professional male golfers were recruited from the Greater Taipei area between August 2020 and July 2021. The age of these golfers ranged from 23 to 60 (mean age = 42.2 ± 10.4) years, and they had a mean experience of 11.2 (standard deviation [SD] = 7.3) years playing golf and a mean handicap score of 16.4 (SD = 7.2). According to United States Golf Association (USGA) statistics, a handicap score of 15 reflects golf skills above 42.58% of male golfers (United States Golf Association, 2024). The sample size was determined based on an a priori power analysis using G^*^Power 3.1. Consistent with a previous electroencephalography study on attentional instruction (Hunt et al., 2013), we set the following input parameters for a paired *t* test: alpha = 0.05, power = 0.80, effect size = 0.33–0.50 (corresponding to ηP^2^ = 0.10–0.20), and actual power = 0.80. We determined the sample size of 34 for this study.

All the recruited participants met the following inclusion criteria: (1) no history of neurological diseases and (2) right-handed. All the participants provided an informed written consent. This study was approved by the Research Ethics Committee of National Taiwan Normal University (201912HM109). All study procedures were carried out in accordance with the relevant guidelines and regulations of the research ethics committee.

### 2.2 Procedure

Participants are required to come to the experiment once and are asked not to consume any beverages containing alcohol or caffeine within 24 hours prior to the test. Upon arriving at the laboratory, written informed consent was obtained from the participants. The participants were then fitted with a Lycra electrode cap (Quick-cap, Neuroscan, Charlotte, NC, USA), and impedance and signals were examined. The resting-state EEG data of the participants were recorded first, followed by a minimum of 12 putting practice trials prior to the formal putting task. EEG signals were recorded during the golf-putting task that consisted of 60 self-paced trials, which were divided into six recording blocks (10 putts per block with an interblock interval of ~2 min). The total duration of the experiment was ~2 h.

### 2.3 Measurements

#### 2.3.1 Golf-putting task

The participants performed a putting task on an indoor artificial golf green with a length of 572 cm and a width of 200 cm. The hole diameter conformed to the standard size (10.8 cm). The distance between the starting point and the hole was 3 m. During an official competition, a golfer's primary goal is to let the ball roll into the hole. Professional golfers, on average, have a one-put probability (success rate) of 40% when they put the ball from a distance of 3 m during the Professional Golfers' Association Tour. This distance determines the results of a golf game due to its medium difficulty. To simulate an actual match, serial variable practice was used by instructing participants to putt from three starting lines at their own pace. Their objective was to let the ball roll into the hole. The task variability was increased to enhance the participants' engagement in the task. A successful putt was defined as the ball rolling into the hole, whereas a failed putt was defined as the ball not rolling into the hole.

#### 2.3.2 SE

For self-efficacy assessment, we used a modified scale based on Turner et al. (2008).

Specifically, participants were requested to their confidence level on a visual analog scale (VAS) ranging from 0 to 10 before each putt, where 0 indicated “not confident at all” and 10 indicated “completely confident”. These were of the form: indicate how confident you believe yourself to be putting task. The advantages of this method are as follows: (1) the psychometric properties of the VAS have received substantial support in previous research (Davey et al., 2007; Hunt et al., 2013) and (2) the VAS can be completed more rapidly and thus reduces interference for participants (Watkins et al., 1994). Based on the median score, individual SE scale scores were classified as high or low SE trials.

#### 2.3.3 Electroencephalography recording and analysis

During the putting task, electroencephalographic (EEG) activity was recorded at 32 sites by using an elastic electrode cap (Quick-Cap, Compumedics Neuroscan, Inc., Charlotte, NC, USA) in accordance with a modified International 10–20 System. Ongoing EEG activity was referenced to the average of the mastoids (A1 and A2), with FPz serving as the ground electrode. Electrooculographic (EOG) activity was recorded using four electrodes placed at the outer canthus of each eye and above and below the left orbit. EEG and EOG signals were recorded using NeuroScan NuAmps software, version 4.5 (Neuroscan), with the bandpass filter ranging from DC to 100 Hz and the notch filter set at 60 Hz. The signals were sampled at a frequency of 1,000 Hz, and the electrode site impedance was maintained below 10 kΩ. Prior to assessing performance, event marker data were collected using an infrared sensor that detected the onset of each putt swing to understand the preparation state (Chen et al., 2022; Wang et al., 2022; Chueh et al., 2023). The swing onset was defined as the event when the participants moved the putter away from the ball to initiate a backswing.

EEG data were processed using EEGLAB software on MATLAB (Delorme and Makeig, 2004). During data processing, EEG signals were filtered using an FIR filter with its digital bandpass ranging from 1 to 50 Hz (6 dB/octave). To focus the independent component analysis (ICA) computation on task-related activity, data exceeding 2 s before and after the event marker were removed. ICA decompositions were performed using the infomax algorithm with default settings in EEGLAB to extract sub-Gaussian components (Bell and Sejnowski, 1995). Subsequently, the icablinkmetrics function (version 3.1) was utilized to eliminate ICA components related to eyeblink artifacts, and EEG signals were reconstructed without these artifacts (Pontifex et al., 2017). The average numbers of IC were excluded (2.68 ± 1.69). Continuous EEG data was then segmented and extracted for a preparatory period of 2 seconds immediately preceding the swing onset for further analysis.

Epochs with amplitudes outside the range of ±150 μV were discarded. The average numbers of bad trial rejected were similar for all the conditions (High-SE =3.68 ± 7.33 and Low-SE = 2.63 ± 6.12).

Artifact-free epochs were fast Fourier transformed with a Hamming window to compute the power spectral density (PSD) of each frequency band with a 0.5-Hz bin. For electrodes, the PSD values of the following regions of interest were averaged: F3, Fz, and F4. The θ power was extracted from the data in the 4–7.5Hz range and The Alpha power was extracted from the data in the 8-13 Hz range. The EEG power data were then naturally log-transformed (ln), as they violated the Shapiro–Wilk normality test (p < 0.05). Performance-related EEG data were analyzed using a previously reported procedure (Chueh et al., 2023).

### 2.4 Statistical analysis

Data were organized and transformed (such as addition, subtraction, and average) using Excel. SPSS (version 23) was used to perform all statistical analyses. Means and SDs were calculated for all data. Based on the median scores of individual SE, each trial was classified into either a high SE trial or a low SE trial. This research applied a separate paired t-test to evaluate the variances in putting success rates and Fmθ across trials with high self-efficacy (SE) and low SE, under the condition that SE, putting success rates, and Fmθ are unrelated. we evaluated the degree of correlation among these three variables. Should there be any correlation, the method will be adjusted to employ ANCOVA. The *t* test was chosen over multiple-factor analysis of variance (ANOVA) to reduce the inflation of type I error from the factors (familywise type I error) (Luck and Gaspelin, 2017, p. 9–11). The α level was set at 0.05, and the effect size was reported as dmatched (Cohen, 2013, p. 351), along with a 95% CI (Moher et al., 2010, Item 17a).

In a control analysis, to determine the task specificity of Fθ in the golf-putting task, ANOVA was used to examine differences in Fθ across different brain regions (FZ, PZ, and CZ). Furthermore, in case of significant findings, a *post hoc* analysis was performed to estimate effect sizes using partial η2 and Cohen's d (for the equation, see Dunlap et al., 1996). In addition, Alpha power has been demonstrated to be a key indicator of focus and engagement, not just in controlled laboratory settings but also when using portable EEG technologies (Arnau et al., 2021). It's crucial to conduct further control analyses to ascertain that the variations observed are, in fact, primarily influenced by theta wave activity.

## 3 Results

**Figure 2:**
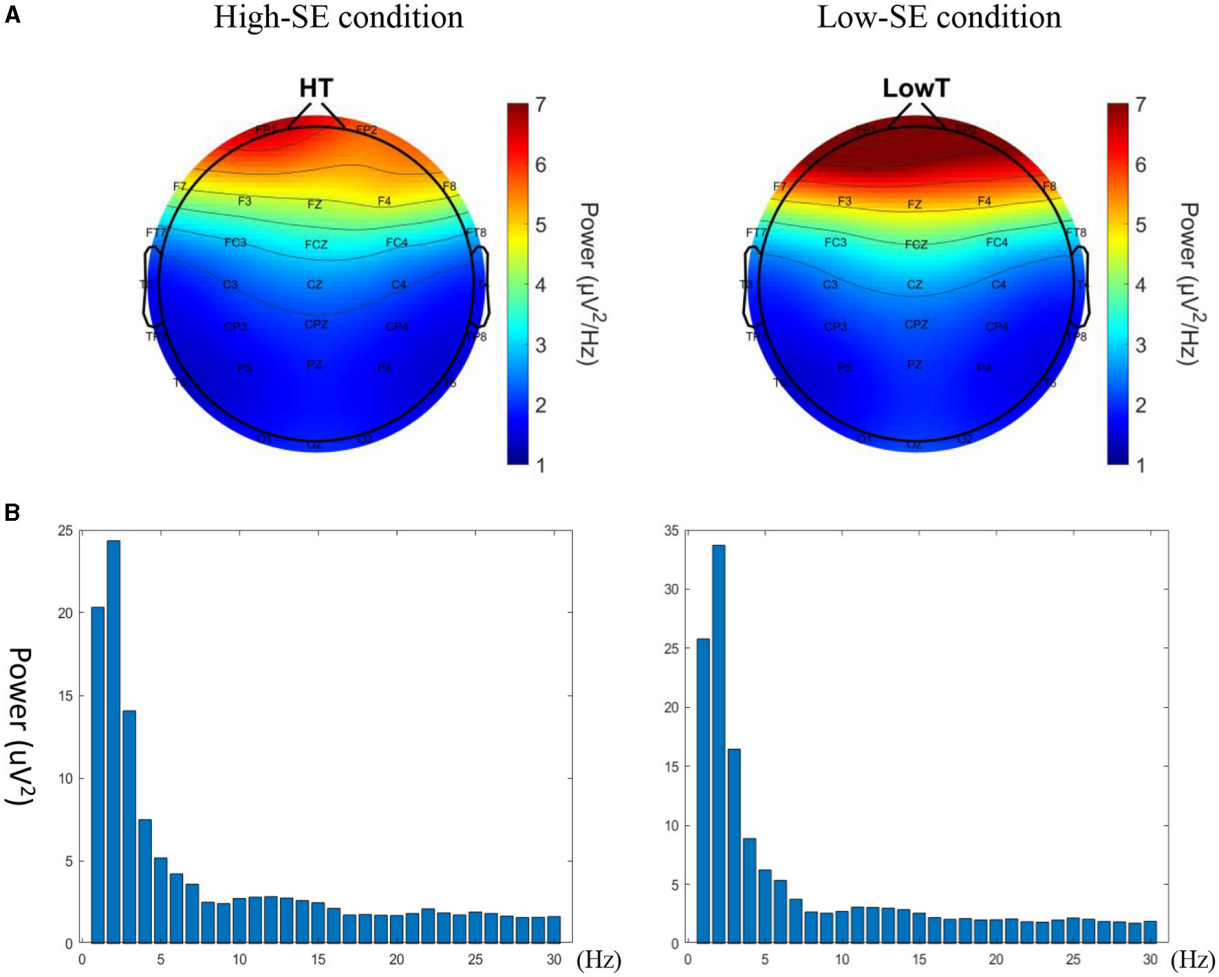
**(A)** θ power between high- and low-SE conditions. **(B)** Power spectrum at Fz between high- and low-SE conditions.

1. A two-pair *t* test (high-SE vs low-SE) was performed to investigate variations in the putting success rate and Fmθ prior to the execution of the putting task. The demographic factors, putting success rate, and EEG findings are listed in Table 1. To rule out some potential competing explanations, we determined the correlation between θ power at Fz and the putting success rate.
2. Putting performance: the mean putting success rate of all the participants was 50.7% ± 15.1%. The putting success rate significantly differed between the high- and low-SE conditions, *t*_(33)_ = 2.97, *p* < 0.01, with the success rate being higher in the high-SE condition than in the low-SE condition. (53.3% > 46.7%).
3. EEG power: the Fmθ value significantly differed between the high- and low-SE conditions, *t*_(33)_ = −2.37, *p* < 0.05, with the Fmθ value being lower in the high-SE condition than in the low-SE condition (4.49 < 5.18) (Figure 2).
4. Control analysis: to check the task specificity of θ in the golf-putting task, ANOVA was used to examine differences (HSE - LSE) in θ across different brain regions (Fz, Cz, and Pz). The ANOVA showed a significant effect of brain regions in θ power, *F*_(1, 32)_ = 3.77, *p* = 0.034, $\eta_{\text{p}}^{2}$ = 0.19, with Fz (−0.61 ± 0.41) having larger differences than Cz (−0.14 ± 0.17) and Pz (−0.14 ± 0.11).

**Table 1:** Summary of behavioral and EEG outcomes.

	**Total**	**High-SE**	**Low-SE**
Putting success rate (%)	50.68 (15.10)	53.32 (17.18)	46.86 (16.96)
SE (scales)	7.99 (1.31)	8.57 (1.27)	7.12 (1.58)
SE trials (/60 trials)	60 (0)	34.5 (12.5)	25.4 (12.4)
Fmθ (μV)	4.85 (4.16)	4.49 (3.93)	5.18 (4.74)
FmAlpha (μV)	2.74 (1.61)	2.66 (1.65)	2.77 (1.69)

**Figure 3:**
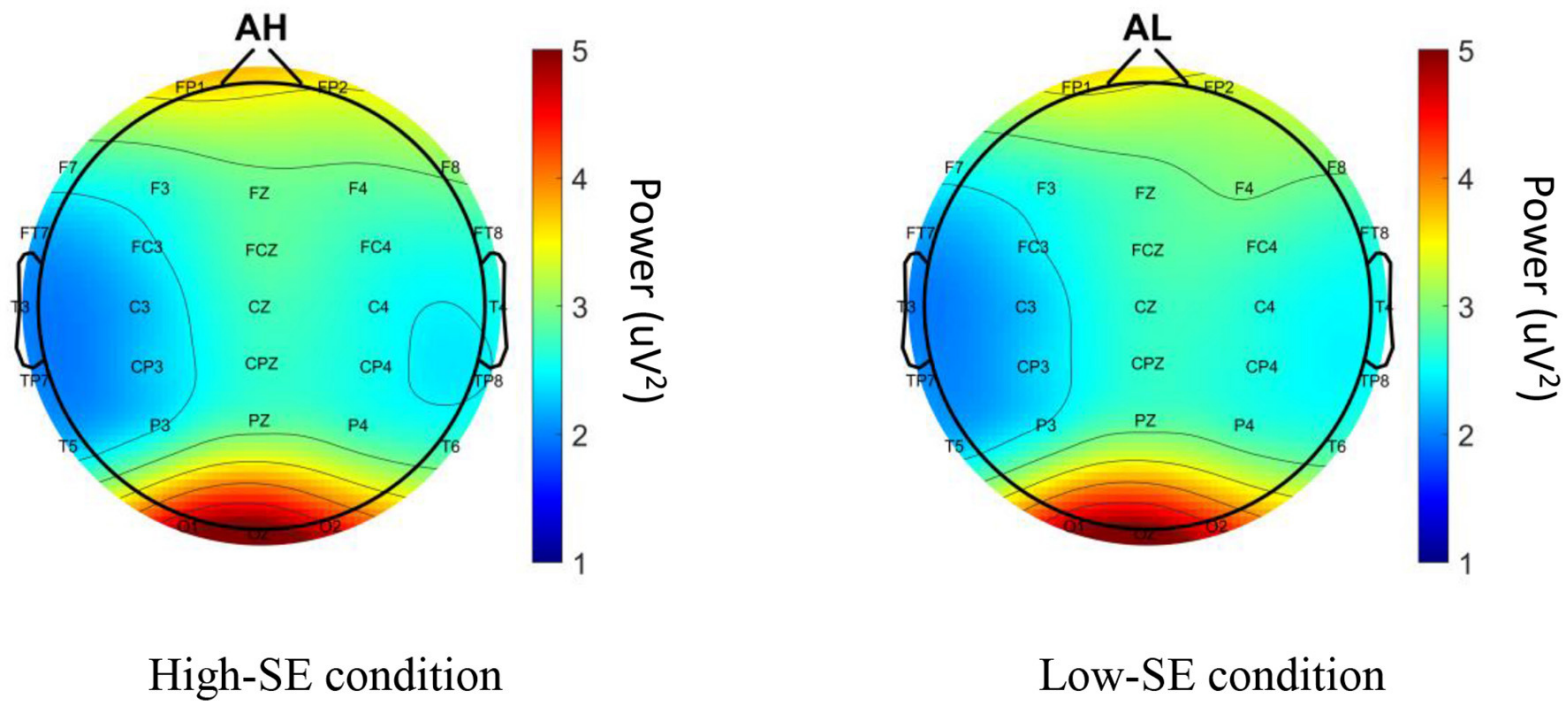
Difference in Alpha power between high- and low-SE conditions.

On the other hand, the Alpha values in both high-SE and low-SE conditions are depicted in Figure 3, yet there are no differences between these conditions. By the side, we didn't find any correlation between θ power at Fz, SE, experience year and the putting success rate.

## 4 Discussion

This study employed electroencephalography to investigate the effect of low and high SE on frontal midline theta and the motor performance. We compared putting performance and EEG power during the preparatory period of a putting task in skilled golfers with high and low SE levels. We observed that the golfers' putting success rate was higher and Fmθ power was lower in higher SE trials.

The results of this study support our hypothesis that golfers with a higher SE score have a higher success rate. This result is consistent with those reported by Chang et al. (2014) and Horcajo et al. (2022). Horcajo et al. (2022) showed that SE was positively related to both physical and cognitive performance. Chang et al. (2014) determined a significant positive correlation between SE and softball throwing performance. To explain the positive relationship between SE and motor performance, Bandura (1990) defined SE as an individual's sense of confidence in their ability to perform a given behavior in various contexts. Bandura described SE as a cognitive mechanism that mediates the relationship between self-appraisal information and an individual's subsequent thoughts, emotions, motivations, and behaviors. SE theory posits that people are more likely to engage in behaviors that they believe they can successfully perform and avoid behaviors in which they feel they will be unsuccessful.

Studies have indicated that self-efficacy (SE) is closely linked with a variety of mechanisms, including cognitive functions, motivation, emotions, and selection processes. Specifically, within the realm of selection processes, research has identified a correlation between SE and cognitive functions, notably in decision-making scenarios. This connection is evident in the realm of sports, where SE has been found to significantly influence decision-making abilities in baseball players, as noted by Hepler and Feltz (2012). Furthermore, from a behavioral perspective, individuals possessing higher levels of SE are more inclined to pursue ambitious goals, as outlined by Bandura (1997), and exhibit superior self-regulation skills (Kane et al., 1996). Considering the impact of factors related to SE, such as decision-making capabilities, goal-setting, self-regulation, and anxiety management, on overall performance, SE emerges as a critical factor for achieving excellence. This has led to its recognition as a key predictor of successful outcomes in various fields (Moritz et al., 2000). The collective body of research underscores the integral role of SE in fostering a framework for individuals to excel, by enhancing their psychological resilience and operational efficiency.

Another primary finding of this study is that the golfers exhibited lower Fmθ power in high-SE trials, supporting the hypothesis that higher SE leads to more automatic processing during golf-putting tasks. This finding is consistent with that of a previous study indicating that individuals with a lower confidence level exhibited increased prefrontal brain activity on functional magnetic resonance imaging (Fleming et al., 2012). Moreover, Hunt et al. (2013) found that the winning group in a shooting competition exhibited a higher confidence level and lower alpha and theta power than the losing group. In contrast, Chatterjee et al. (2021) found higher theta and alpha power in the frontal cortex in high-confidence conditions than in low-confidence conditions; cognitive experiments showed that higher SE is associated with a stronger deployment of attentional control (Frömer et al., 2021). However, it is important to note that the latter study did not specifically examine sports performance. These findings collectively underscore the potential of Fmθ power as a critical intermediary in the complex interplay between SE and athletic performance. The consistency across these studies suggests that SE not only affects psychological states but also has a tangible impact on physiological responses during competitive sports. This underscores the importance of developing strategies to boost athletes' confidence as a means to improve their focus, reduce unnecessary cognitive load, and enhance overall performance.

This study used EEG evidence to determine sports performance, focusing on the expert paradigm in self-paced, precision sports. A previous meta-analysis indicated a nonsignificant increase in alpha activity and a decrease in theta activity in this context (Filho et al., 2021). The “relaxed brain” neural marker is characterized by increased alpha activity across the cortex, which inhibits brain areas unrelated to the task at hand, particularly in the frontal lobe where the highest alpha and lowest theta activity are found (Filho et al., 2021). These findings support those of previous studies demonstrating that a relaxed and focused brain is essential for optimal sports performance (Pacheco, 2016; Bertollo et al., 2020; Hatfield et al., 2020). Although theta activity may indicate the need and timing of cognitive control, it may not necessarily play a functional role in downstream signaling. Experienced performers can enhance their motor skills by suppressing irrelevant cognitive and motor processes; this phenomenon is known as neuromotor noise. The brain needs to integrate cognitive control processes into sensorimotor systems to achieve behavioral control. The suppression of neuromotor noise is a crucial factor in the development of enhanced motor skills, and frontal theta is involved in sensorimotor integration (Cruikshank et al., 2012). Thus, the findings of this study indicated that SE affects the electrophysiological state of the brain, leading to more automated actions.

Our findings support and extend Bandura's SE theory by demonstrating that the anticipation of outcomes may affect the automation of actions and that Fmθ power may mediate the relationship between SE and sports performance. Optimal sports performance is characterized by a constant focus on the present as well as physical and psychological relaxation, which enable effortless automatic movements. Fmθ is among the most crucial elements of optimal sports performance (Williams and Krane, 2020).

Fmθ power measures attentional allocation to achieve a desired cognitive–motor behavior, particularly as measured from the anterior region of the scalp, and is indicative of task-relevant working memory processes (Jensen and Tesche, 2002; Sauseng et al., 2010). Studies have suggested that cognitive control originates in the frontal cortex and is mediated by cortical oscillations that underlie long-range communication in the brain (Buzsáki et al., 2012; Cavanagh and Frank, 2014). The level of Fmθ was proportional to the degree of effort invested in response inhibition and preparation (Isabella et al., 2019). Frontal theta oscillations might be involved in physiological mechanisms underlying cognitive control because they increase during working memory (Jensen and Tesche, 2002; Zakrzewska and Brzezicka, 2014), mental arithmetic (Gärtner et al., 2015), response preparation (Womelsdorf et al., 2010), affective regulation (Cavanagh and Shackman, 2015), top–down attention (Cavanagh and Frank, 2014). A lower level of Fmθ may suggest that golfers were not engaged in active mental control during the putting task. This speculation is supported by two previous studies (Kao et al., 2013, 2014), which have reported that superior putting performance was preceded by a lower level of Fmθ (Kao et al., 2013) and that one neurofeedback training session on reducing Fmθ power effectively improved the putting performance of highly skilled golfers (Kao et al., 2014). Individuals with elevated levels of SE often lean toward their first, instinctive reactions, considering fewer options in the process. To achieve behavioral control, the brain integrates cognitive control processes into sensorimotor systems (Hepler and Feltz, 2012). The modulation of behavioral responses through cognitive regulation is frequently accompanied by theta-band activity in the frontal cortex, where Fmθ power serves as an indicator of attentional allocation toward achieving desired cognitive–motor responses (Sauseng et al., 2010). SE, as established through the prediction of consequences, might influence the automatization of actions and Fmθ power before the act of putting, thus potentially mediating the association between SE and sports performance.

Although this study using the VAS method yielded novel insights into the putting performance of golfers based on EEG findings, it is not without limitations. Firstly, factors such as learning, fatigue, and variable practice may affect putting performance. Thus, future studies should consider incorporating relevant subjective measurements such as self-report, VAS when designing their experiments. Nevertheless, we addressed some of these limitations by providing an opportunity for practice before the primary task, including a 2-minute rest period between blocks, and setting three starting lines for putting (serial variable practice). However, the additional analysis indicated no significant difference [*t* (26) = −0.815, *p* =0.422] in the putting success rate between the first and last three blocks was observed. We suggested that these factors exerted only a negligible impact on putting performance in this study. Secondly, this study employed a putting task at a distance of 3 m, with medium difficulty, and categorized performance into binary variables (i.e., successful and unsuccessful putt) based on the reality of golf games and the purpose of this study. Future studies should consider using a longer distance (e.g., professional golfers' average two putts from 33 feet, see Broadie, 2012) for the putting task and measure performance outcomes as continuous variables, such as the radial error (i.e., the distance between the hole and ball), to gain a more comprehensive understanding of the cortical signatures of superior performance.

To summarize, this study contributes to understanding of the neurophysiological mechanisms underlying the effects of SE on sports performance and extends to SE theory (Bandura, 1997; Moritz et al., 2000). SE determined by the anticipation of outcomes, may influences the automation of movements and the activity of frontal midline theta (Fmθ) power before executing a putting action. This relationship highlights SE's role in bridging cognitive expectations with physical performance in sports, emphasizing how psychological Self-efficacy could shape sport performance and execution efficiency. Moreover, our findings indicate that higher individual SE is associated with improved putting performance and greater automation characteristics in golfers. Therefore, enhancing SE before putting can be a promising strategy for improving overall sports performance in golfers.

## Data availability statement

The datasets presented in this study can be found in online repositories. The names of the repository/repositories and accession number(s) can be found below: This study was not preregistered. All data and measures used in the study are publicly available under the Open Science Framework (https://osf.io/x5vsg).

## Ethics statement

This study was approved by the Research Ethics Committee of National Taiwan Normal University (201912HM109). All study procedures were carried out in accordance with the relevant guidelines and regulations of the research ethics committee. The studies were conducted in accordance with the local legislation and institutional requirements. The participants provided their written informed consent to participate in this study.

## Author contributions

C-LY: Writing – original draft, Visualization, Project administration. C-WK: Writing – original draft, Data curation, Conceptualization. J-HW: Writing – review & editing, Methodology, Formal analysis. EH: Writing – review & editing, Investigation, Data curation. W-CC: Writing – review & editing, Project administration, Data curation. R-TY: Writing – review & editing, Project administration, Data curation. K-PW: Writing – review & editing, Supervision, Conceptualization. T-MH: Writing – review & editing, Supervision, Funding acquisition, Conceptualization.
